# Volume replacement with Ringer-lactate is detrimental in severe hemorrhagic shock but protective in moderate hemorrhagic shock: studies in a rat model

**DOI:** 10.1186/cc13182

**Published:** 2014-01-06

**Authors:** Bjoern Hussmann, Sven Lendemans, Herbert de Groot, Ricarda Rohrig

**Affiliations:** 1 Trauma Surgery Department, University Hospital Essen, Hufelandstraße 55, Essen 45122, Germany; 2 Institute of Physiological Chemistry, University Hospital Essen, Hufelandstraße 55, Essen 45122, Germany

## Abstract

**Introduction:**

To date, there are insufficient data demonstrating the benefits of preclinically administered Ringer-lactate (RL) for the treatment of hemorrhagic shock following trauma. Recent animal experiments have shown that lactate tends to have toxic effects in severe hemorrhagic shock. This study aimed to compare the effects of RL administered in a rat model of severe hemorrhagic shock (mean arterial blood pressure (MAP): 25 to 30 mmHg) and moderate hemorrhagic shock (MAP: 40 to 45 mmHg).

**Methods:**

Four experimental groups of eight male Wistar rats each (moderate shock with Ringer-saline (RS), moderate shock with RL, severe shock with RS, severe shock with RL) were established. After achieving the specified depth of shock, animals were maintained under the shock conditions for 60 minutes. Subsequently, reperfusion with RS or RL was performed for 30 minutes, and the animals were observed for an additional 150 minutes.

**Results:**

All animals with moderate shock that received RL survived the entire study period, while six animals with moderate shock that received RS died before the end of the experiment. Furthermore, animals with moderate shock that received RL exhibited considerable improvements in their acid-base parameters and reduced organ damage.

In contrast, in animals with severe shock, only two of the animals receiving RS survived but all of the animals receiving RL died early, before the end of the study period. Moreover, the severe shock animals that were treated with RL exhibited considerably worsened acid-base and metabolic parameters.

**Conclusions:**

The preclinical use of RL for volume replacement has different effects depending on the severity of hemorrhagic shock. RL exhibits detrimental effects in cases of severe shock, whereas it has pronounced protective effects in cases of moderate shock.

## Introduction

Accidents remain a major cause of death. Apart from severe traumatic brain injury, uncontrolled bleeding and corresponding hemorrhagic shock play significant roles in mortality [1-3]. Except for patient positioning (for example, 30° semi-recumbent position), little preclinical management of severe craniocerebral trauma is possible; this condition is the most common cause of premature mortality. In contrast, hemorrhagic shock can be managed to prevent fatality. Hess *et al*. demonstrated that bleeding in hemorrhagic shock and its resulting consequences are the most common preclinical avoidable cause of death [4]. The sequelae of hemorrhagic shock that generally occur later after injury, while patients are still in the hospital, such as single- or multi-organ failure, are the most important factors in death after severe trauma with bleeding [1].

In addition to the top priority defined by the Advanced Trauma LifeSupport (ATLS®), that is, to “stop the bleeding”, volume replacement is typically performed during the preclinical course of hemorrhagic shock treatment by administering crystalloid solutions to maintain microcirculation [5-8].

Many studies, and in particular, animal experiments, have shown that resuscitation with lactate-containing solutions improves hemodynamic parameters, blood coagulation and survival compared to resuscitation using crystalloid solutions without metabolizable anions [9-11]. However, lactate-containing solutions may alter plasma lactate concentrations, which are used as a marker of hypoxemia, and solutions containing both L-lactate and D-lactate may induce pulmonary and hepatic apoptosis [12-15].

Contrary to recently published results, our group has shown that Ringer-lactate (RL) may mediate toxic effects in rats with severe hemorrhagic shock (mean arterial blood pressure (MAP) 25 to 30 mmHg) [16]. However, whether this effect occurs exclusively in severe hemorrhagic shock remains unclear. In severe hemorrhagic shock, energy production is based mainly on anaerobic metabolism, during which lactate may accumulate. Therefore, the objective of this study was to investigate the effects of reperfusion with RL on survival, organ damage, hemodynamics and acid-base balance in rats experiencing two different levels of hemorrhagic shock (MAP 25 to 30 mmHg and 40 to 45 mmHg).

The composition of Ringer-saline (RS) is highly comparable with that of Ringer-lactate (RL) except for chloride, which replaces the metabolizable anion lactate. Due to this similarity, RS seems to be the appropriate solution to study the effects of lactate. Although RS is rarely used clinically, in addition to sodium and chloride, it contains potassium and calcium ions, thus appearing to be the more physiological solution, which is another reason for the use of RS instead of, for example, normal saline as a control in this study (Table 1).

**Table 1:** Composition of Ringer-saline (RS) and Ringer-lactate (RL)

	**RS**	**LR**
NaCl	8.6 g/l	6.00 g/l
Na-Lac	—	0.34 g/l
KCl	0.30 g/l	0.42 g/l
CaCl_2_	0.33 g/l	0.27 g/l
Na^+^	147.2 mM	131 mM
Cl^-^	155.7 mM	112 mM
K^+^	4 mM	5.6 mM
Ca^2+^	2.25 mM	1.84 mM
Lac	—	28.3 mM
Osmolarity	309 mOsm/l	278 mOsm/l

Lac, lactate; Na-Lac, sodium lactate.

## Materials and methods

### Chemicals/materials

RS and RL were obtained from Fresenius (Bad Homburg, Germany), ketamine 10% was obtained from Ceva (Düsseldorf, Germany), lidocaine (Xylocaine 1%) was obtained from AstraZeneca (Wedel, Germany), acid citrate dextrose-A (ACD-A) solution was obtained from Baxter (Deerfield, IL, USA) and Portex catheters (inner diameter: 0.58 mm, outer diameter: 0.96 mm) were obtained from Smiths Medical International (Hythe, UK).

### Animals

Male Wistar rats (400 to 450 g) were obtained from the central animal unit of the Essen University Hospital. Animals were kept under standardized temperature (22 ± 1°C) and humidity (55 ± 5%) conditions with a 12-h:12-h light:dark cycle. Animals were fed *ad libitum* (ssniff-Spezialdiäten, Soest, Germany) with free access to water and were not fasted prior to the experiments. All animals received ethical care according to the standards of Annex III of directive 2010/63/EU of the European Parliament and of the Council of 22 September 2010 on the protection of animals used for scientific purposes [17]. The experimental protocol was approved by the North Rhine-Westfalia State Office for Nature, Environment, and Consumer Protection (Landesamt für Natur, Umwelt, und Verbraucherschutz Nordrhein Westfalen), Germany, based on the local animal protection act.

### Anesthesia, analgesia and surgical procedures

The resuscitation schedule and anesthesia, analgesia, catheter insertions, shock induction, blood sampling and organ resection were performed as described previously [18], with minor modifications. Rats were anesthetized with isoflurane (2% in 100% medical O_2_ at 4 l/minute for anesthesia induction; 1% to 1.5% at 1 l/minute throughout the experiment) using face masks connected to a vaporizer (Isoflurane Vet. med. Vapor; Dräger, Lübeck, Germany) and received ketamine (50 mg/kg, subcutaneously (s.c.)) administered into the right chest wall for analgesia. Lidocaine (5 mg/kg, s.c.) was administered into the right groin before a skin-deep incision was made for catheter insertion. Subsequently, a Portex catheter was placed into the femoral artery, and an identical catheter was placed into the femoral vein. Each catheter was held in place with surgical sutures.

### Induction of hemorrhagic shock and resuscitation regimen

The animals were allowed to adapt to the catheter insertion before hemorrhagic shock induction. Shock was induced by removing 2 ml blood every three minutes through the femoral artery catheter using a 2-ml syringe (Terumo, Leuven, Belgium). The first syringe was prefilled with 0.2 ml acid citrate dextrose-A solution (ACD-A). The syringe with citrated blood was stored at 37°C and used to adjust shock severity, if needed. Bleeding was continued until the MAP dropped to 1) 25 to 30 mmHg (severe shock) or 2) 40 to 45 mmHg (moderate shock); this process typically required approximately 20 minutes. Because all animals exhibited a similar weight (400 to 450 g) and total blood volume is dependent on body weight in this range, the shed blood volume in the groups to be compared was nearly identical. The blood volume withdrawn in the severe shock groups was about 12 to 14 ml, whereas it was around 6 to 8 ml in the moderate shock groups. For the next 60 minutes, the MAP was maintained, typically without requiring further intervention. In some cases, however, small amounts (0.1 to 0.5 ml aliquots) of the citrated blood were administered, or additional blood (0.1 to 0.5 ml aliquots) was withdrawn to maintain the MAP within the desired range. After the shock phase, study group-specific resuscitation fluids were infused via the femoral vein using a syringe pump (Perfusor-Secura FT; B Braun, Melsungen, Germany), requiring approximately 30 minutes. Experiments were continued for another 150 minutes or until the rat died. To compensate for fluid loss via surgical areas and the respiratory epithelium, 0.9% NaCl solution (5 ml/kg × h, 37°C) was infused through the femoral vein catheter throughout the entire experiment [18].

### Experimental groups

All 38 animals were randomly assigned to the following groups:

– sham group (no shock, no resuscitation, six animals)

– moderate shock/RS group (40 to 45 mmHg MAP, resuscitation with RS equivalent to three times the shed blood volume, eight animals)

– moderate shock/RL (40 to 45 mmHg MAP, resuscitation with RL equivalent to three times the shed blood volume, eight animals)

– severe shock/RS group (25 to 30 mmHg MAP, resuscitation with RS equivalent to three times the shed blood volume, eight animals)

– severe shock/RL group (25 to 30 mmHg MAP, resuscitation with RL equivalent to three times the shed blood volume, eight animals)

The fluid volume used for resuscitation was based on the well-known 3:1 rule [19].

### Biomonitoring

Systolic blood pressure, diastolic blood pressure and MAP were recorded continuously via the femoral artery catheter, which was connected to a pressure transducer, and displayed on a monitor. RS was delivered at a rate of 3 ml/h to maintain catheter functionality. The heart rate was determined using systolic blood pressure spikes. The core body temperature was continuously monitored using a rectal probe. Cooling was prevented with the use of a heated operating table and by covering the animals with aluminum foil. Oxygen saturation was recorded continuously using a pulse oximeter (OxiCliq A; Nellcor, Boulder, CO, USA) placed at the left hind limb. The breathing rate was determined in 10-minute intervals based on the number of ventilation movements within 15 sec.

### Assessment of blood and plasma parameters

A 2-ml syringe containing 80 IU electrolyte-balanced heparin (Pico50; Radiometer Medical, Brønshøj, Denmark) was used to collect blood samples (0.7 ml) from the femoral artery catheter for blood gas analysis and for the assessment of marker enzyme activities. Blood was collected immediately after syringe insertion (T = 0 minute), after the insertion of all catheters (T = 10 minutes), at the end of shock induction (T = 40 minutes), immediately before initiating resuscitation (T = 100 minutes), at the end of resuscitation (T = 130 minutes), and during the final observation phase (T = 160 minutes, 220 minutes and 280 minutes). After each blood sample was drawn, the animal was given a 0.7 ml bolus of RS via the femoral artery (with the additional effect of flushing the catheter and maintaining its functionality). Arterial blood pH, oxygen and carbon dioxide partial pressures (pO_2_, pCO_2_); oxygen saturation; base excess (BE); hemoglobin concentration; hematocrit; electrolytes (Na^+^, K^+^, Cl^-^, Ca^2+^); metabolic parameters (lactate and glucose concentrations); and osmolality were assessed using a blood gas analyzer (ABL 715; Radiometer, Copenhagen, Denmark). Blood plasma was obtained by centrifugation at 3,000 × *g* for 15 minutes at 25°C, stored at 4°C, and used within 4 h. The plasma activity of lactate dehydrogenase (LDH), as a general marker for cell injury; aspartate aminotransferase (AST) and alanine aminotransferase (ALT), as markers for liver cell injury; creatine kinase (CK), as a marker for muscle cell injury; and plasma creatinine concentration, as a marker of renal function, were determined with a fully automated clinical chemistry analyzer (Vitalab Selectra E®; VWR International, Darmstadt, Germany).

### Statistics

Experiments were performed with eight animals per experimental group, except for the sham group, which consisted of six animals. Data are expressed as the mean values ± SEM. Outliers were removed after box-plot analysis. Multiple group comparisons of the post-resuscitation phase were performed using one-way analysis of variance (ANOVA) for independent or repeated measures. ANOVAs were followed by Fisher’s Least Significant Difference (LSD) *post-hoc* analysis. Survival curves were generated according to the Kaplan-Meier method and were compared with the log rank test. *P*-values <0.05 were considered significant.

## Results

### Survival

**Figure 1:**
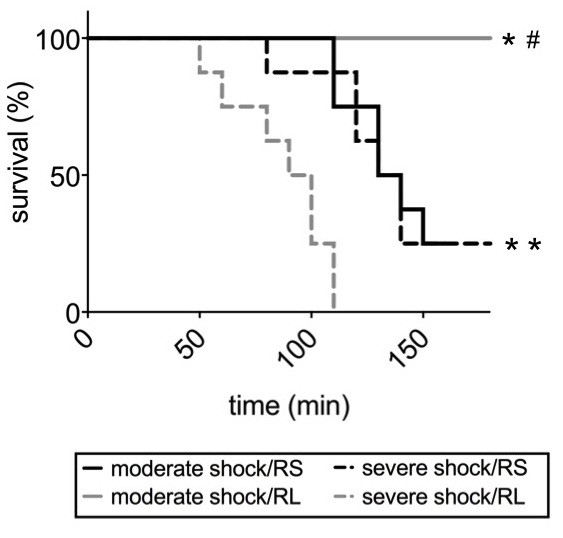
**Effects of Ringer-saline (RS) and Ringer-lactate (RL) on survival after shock.** Rats were subjected to moderate or severe hemorrhage, resuscitated with either Ringer-saline (RS) or Ringer-lactate (RL), and then observed until all animals of the shock/RS and the shock/RL group died (between T = 50 and T = 150 minutes). The survival of each group is shown as a Kaplan-Meier plot (n = 8, logrank *P <0.05; vs. severe shock/RL, # *P* <0.05 vs. severe and moderate shock/RS groups).

All animals of the sham group survived (not shown). The same was true for all animals of the moderate shock/RL group (Figure 1). In contrast, only two animals of the moderate shock/RS group survived the entire study period. The earliest death in this group occurred at 110 minutes after resuscitation, that is, at T = 240 minutes of the overall experimental time.

The earliest death in the severe shock/RS group occurred 80 minutes after resuscitation, but two animals survived the entire study period. All rats in the severe shock/RL group died within 110 minutes after resuscitation. The earliest death in this group occurred 50 minutes after resuscitation, that is, at T = 180 minutes.

### Hemodynamics and other systemic parameters

**Figure 2:**
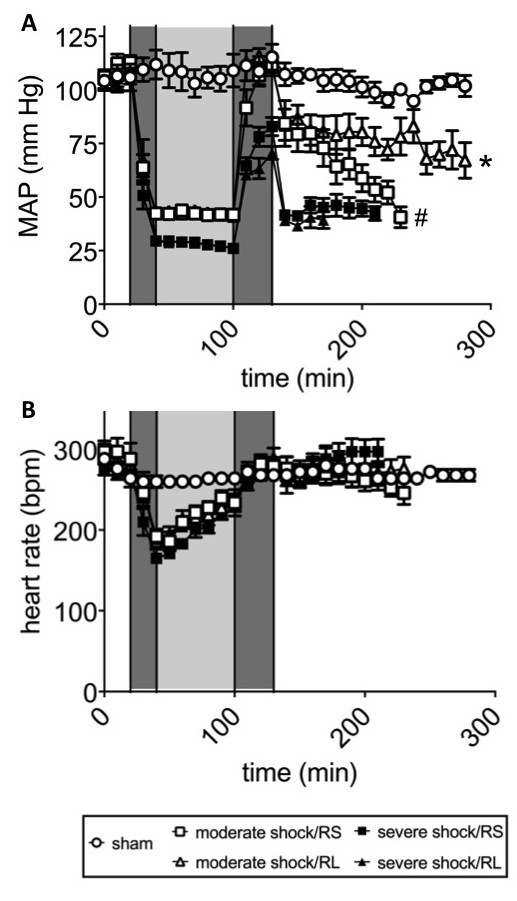
**Effects of Ringer-saline (RS) and Ringer-lactate (RL) on blood pressure and heart rate after shock.** Rats were subjected to moderate or severe hemorrhage (shock induction: dark gray; shock phase: light gray), resuscitated (dark gray) with either Ringer-saline (RS) or Ringer-lactate (RL), and observed until the end of the experiment or until the first animal of each comparison group died. **(A)** Mean arterial blood pressure (MAP) and **(B)** heart rate (HR; bpm, beats per minute). The results are shown as the mean values ± SEM (n = 8 animals per shock group, n = 6 sham group animals). SEM values that are not visible are located behind the symbols. One-way repeated-measures ANOVA with Fisher’s Least Significant Difference (LSD) *post-hoc* test were performed with * *P* <0.05 (vs. all other shock groups); # *P* <0.05 (vs. severe shock groups).

In the sham group, the MAP remained at approximately 105 mmHg (Figure 2A). In the moderate shock groups, the MAP was lowered to 40 to 45 mmHg within 20 minutes of shock induction and maintained in this range for 60 minutes. Upon resuscitation, the MAP returned to an average level of 110 mmHg in the animals of both moderate shock groups. Following resuscitation, the MAP of the moderate shock/RS group continuously decreased to approximately 35 mmHg at the end of the experiment. In the moderate shock/RL group, the MAP only slightly decreased after resuscitation and remained at approximately 70 mmHg for the remainder of the experiment.

In the severe shock groups, the MAP was reduced to 25 to 30 mmHg within 20 minutes of shock induction and maintained at this level for 60 minutes (Figure 2A). Upon resuscitation, the MAP recovered to approximately 75 mmHg (in the severe shock/RS group) and 65 mmHg (in the severe shock/RL group). In the post-resuscitation phase, the MAP rapidly dropped to approximately 30 mmHg and remained at this level until the death of the animals.

The heart rates of the animals in the sham group remained stable at approximately 270 beats per minute (bpm; Figure 2B). In all shock groups, the heart rate decreased to approximately 180 bpm during shock induction and recovered progressively during the shock period and the resuscitation phase. In the moderate shock/RS and moderate shock/RL groups, the heart rates increased to 250 bpm and remained at this level in the post-resuscitation phase. In the severe shock/RS and severe shock/RL groups, heart rates of approximately 300 bpm were observed in the post-resuscitation phase.

The mean breathing rate of the sham group animals was approximately 50 breaths per minute (data not shown). In all animals of the shock groups, the respiratory rate was approximately 60 breaths per minute during shock induction, the shock period and the post-resuscitation phase (data not shown).

The core body temperature of the sham group animals remained at approximately 37°C throughout the experiment (data not shown). In contrast, the temperature of all shock group animals decreased by almost 1°C during shock induction and in the shock phase and returned to 37°C upon resuscitation.

### Plasma electrolyte concentration

Plasma Na^+^, Ca^2+^ and K^+^ concentrations remained almost constant in all groups (Table 2). The plasma Cl^-^ concentrations of both shock groups that received RS were significantly greater than those of the sham control and the RL-receiving groups.

**Table 2:** Effects of Ringer-saline (RS) and Ringer-lactate (RL) on hematocrit and plasma electrolytes after shock

	**Sham**	**Sham**	**Severe shock/RS**	**Severe shock/RL**	**Moderate shock/RS**	**Moderate shock/RL**
**Parameter**	*T = 20 minutes*	*T = 280 minutes*	*T = 170 minutes*	*T = 170 minutes*	*T = 230 minutes*	*T = 230 minutes*
**Hematocrit (%)**	40.2 ± 0.24	32.01 ± 0.86	21.2 ± 0.7	21.3 ± 0.7	23.3 ± 1.2	23.8 ± 1.0
**Na**^**+ **^**(mmol/l)**	138.8 ± 0.3	138.5 ± 0.5	141.1 ± 0.8	138.3 ± 0.9	140.7 ± 1.2	138.8 ± 0.8
**Ca**^**2+ **^**(mmol/l)**	1.4 ± 0.01	1.4 ± 0.02	1.5 ± 0.03	1.5 ± 0.02	1.4 ± 0.04	1.4 ± 0.03
**K**^**+ **^**(mmol/l)**	5.1 ± 0.05	5.5 ± 0.2	5.6 ± 0.1	5.6 ± 0.2	5.9 ± 0.3	5.6 ± 0.2
**Cl**^**- **^**(mmol/l)**	105 ± 0.6	111 ± 0.9	121 ± 0.6	117 ± 0.9*#	122 ± 2.3	116 ± 1.5*#

Rats were subjected to moderate or severe hemorrhage and then resuscitated with either Ringer-saline (RS) or Ringer-lactate (RL). Electrolyte and hematocrit concentrations were measured before shock induction (baseline, T = 10 minutes), at the end of the experiment (sham group; shock/RS, shock/RL; T = 280 minutes) or at T = 150 minutes (that is, the last blood sample taken before the earliest death in the groups to be compared, shock/RS and shock/RL). The baseline values of the sham group were not significantly different from those of the other groups. Values are shown as the means ± SEM. *P <0.05 (vs. moderate shock/RS); # *P* <0.05 (vs. severe shock/RS).

### Acid**-**base and metabolic status

**Figure 3:**
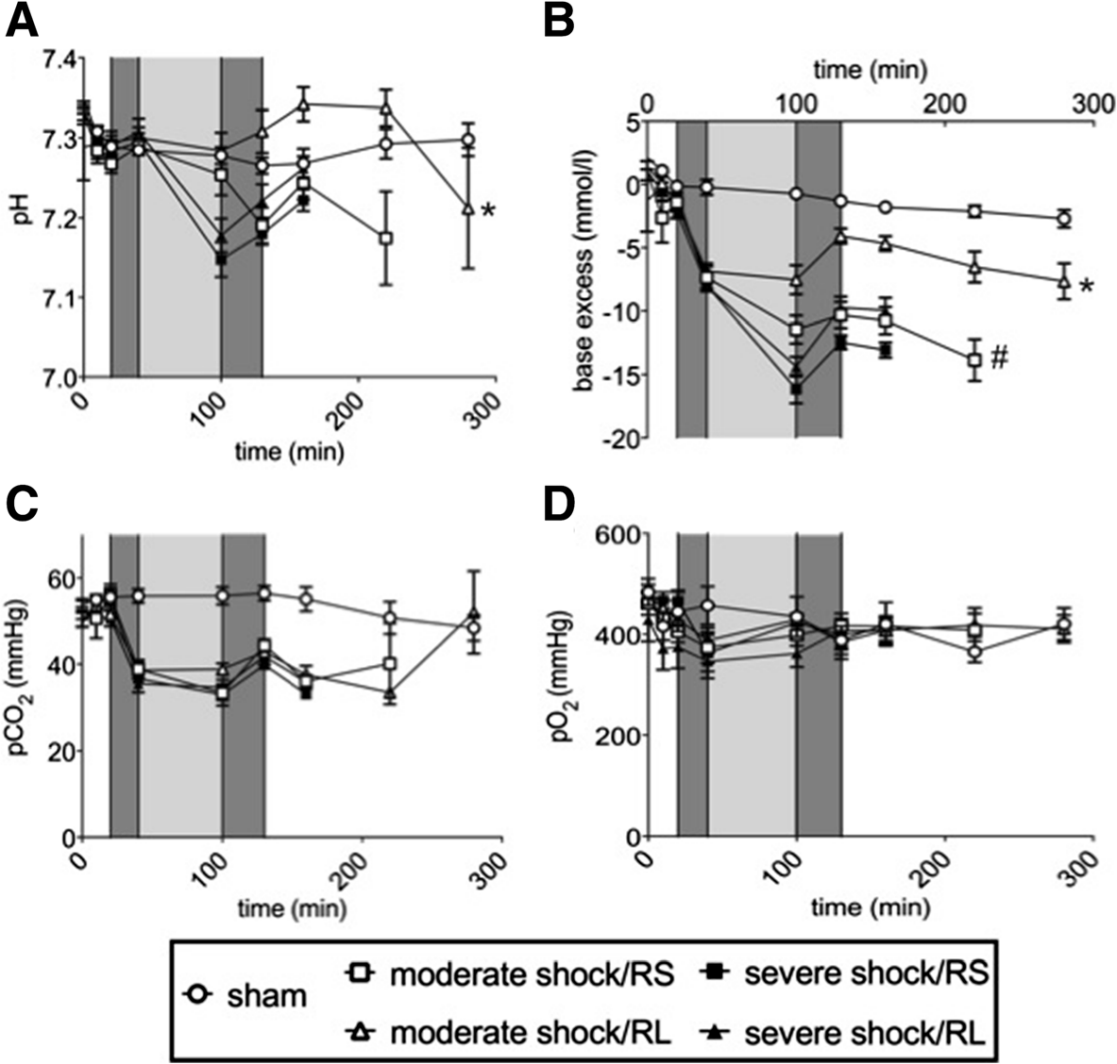
**Effects of Ringer-saline (RS) and Ringer-lactate (RL) on the acid–base equilibrium and pO**_**2 **_**after shock.** Rats were subjected to moderate or severe hemorrhage (shock induction: dark gray; shock phase: light gray), resuscitated (dark gray) with either Ringer-saline (RS) or Ringer-lactate (RL), and observed until the end of the experiment or until the first of the animals of the comparison groups died. Blood gas analysis was performed at distinct time intervals, with T = 280 minutes representing the last possible point of comparison. The following parameters were determined in arterial blood samples at the indicated time points: **(A)** blood pH, **(B)** base excess, **(C)** CO_2_ partial pressure (pCO_2_) and **(D)** O_2_ partial pressure (pO_2_). Values are presented as the means ± SEM (n = 8 animals per shock group, n = 6 sham group animals). SEM values that are not visible are located behind the symbols. *P <0.05 (vs. all other shock groups), # *P* <0.05 (vs. severe shock/RS group).

In the sham group, the blood pH, base excess and pCO_2_ values remained almost constant at 7.3, -2 mmol/l and 55 mmHg, respectively (Figure 3A-C). In the severe shock and moderate shock groups, metabolic acidosis was observed, as indicated by reduced pH and/or base excess, which was partially compensated by a decrease (normalization) in pCO_2_. Metabolic acidosis was most pronounced at the end of the shock phase and recovered partially, but only temporarily, upon resuscitation and during the post-resuscitation phase. The most acidic pH (below 7.2) and the lowest base excess values (−15 mmol/l) were observed in animals of the severe shock groups, with minor differences between the severe shock/RS and severe shock/RL group. In the moderate shock/RS group, metabolic acidosis remained pronounced (base excess −11 mmol/l at the end of the shock phase), while in the moderate shock/RL group only slight metabolic acidosis with almost normal pH values was observed.

The pO_2_ varied between 350 and 490 mmHg with no significant differences within or among the study groups (Figure 3D).

**Figure 4:**
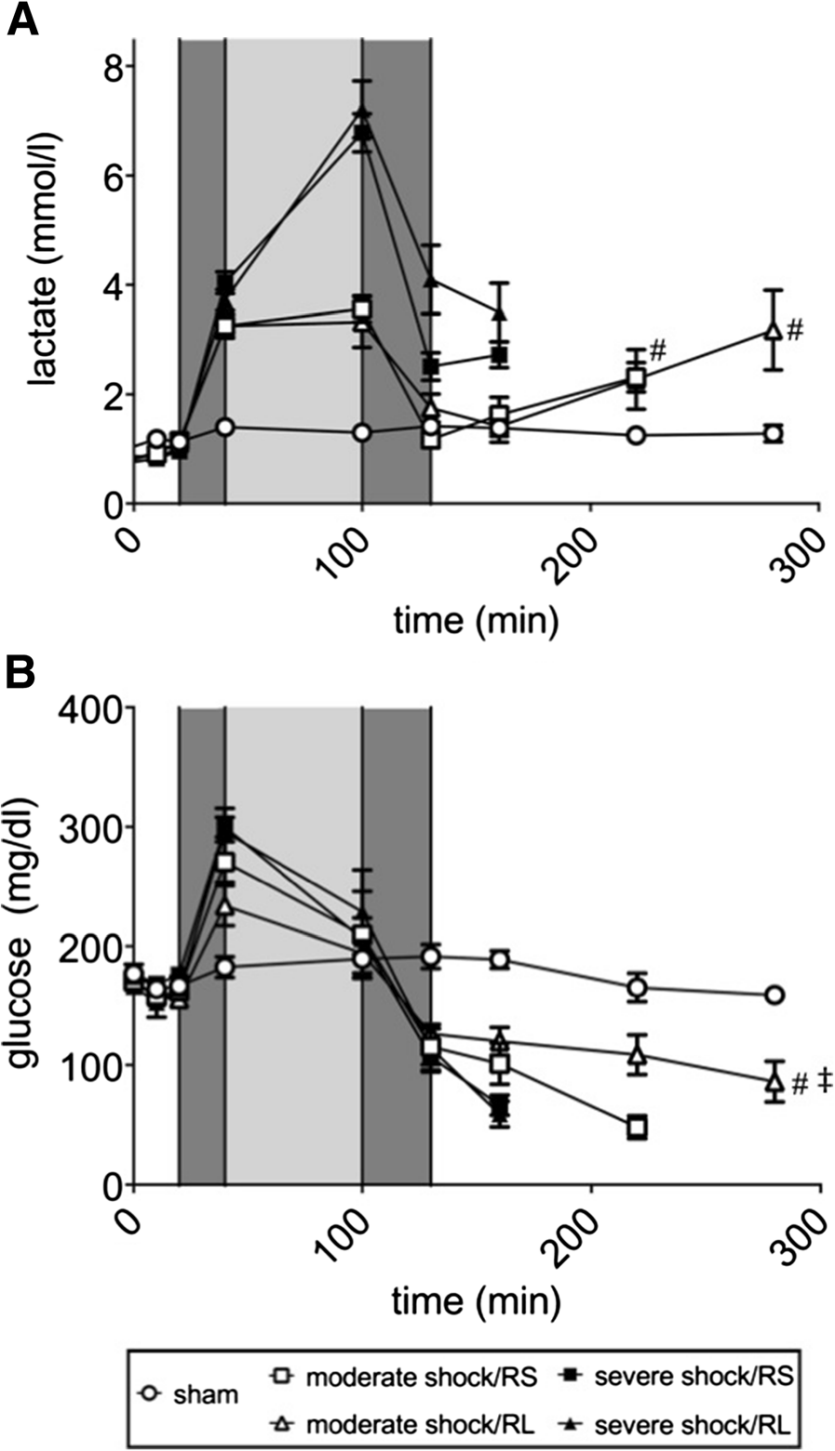
**Effects of Ringer-saline (RS) and Ringer-lactate (RL) on blood lactate and glucose concentration after shock.** Rats were subjected to moderate or severe hemorrhage (shock induction: dark gray; shock phase: light gray), resuscitated (dark gray) with either Ringer-saline (RS) or Ringer-lactate (RL), and observed until the end of the experiment or until the first of the animals of the comparison groups died. Blood gas analysis was performed at distinct time points, with T = 280 minutes representing the last possible point of comparison. Blood lactate **(A)** and glucose concentrations **(B)** were measured at the indicated time points. Values are presented as the means ± SEM (n = 8 animals per shock group, n = 6 sham group animals). SEM values that are not visible are located behind the symbols. One-way repeated-measures ANOVA and Fisher’s Least Significant Difference (LSD) *post-hoc* test were performed. # *P* <0.05 (vs. severe shock groups); ‡ *P* <0.05 (vs. moderate shock/RS group).

The blood lactate concentration remained below 1.5 mmol/l in the sham group (Figure 4A). Consistent with the results indicating shock-induced metabolic acidosis, blood lactate rapidly increased to approximately 4 mM after shock induction. In the moderate shock groups, the blood lactate concentrations remained at this level during the shock phase, whereas those of the severe shock groups increased further, to 7 mmol/l. Upon resuscitation, the blood lactate concentrations promptly decreased to 1 mmol/l in both moderate shock groups and to 2.5 and 4 mmol/l in the severe shock/RS and severe shock/RL groups, respectively. Lactate levels remained roughly constant (in the severe shock groups) or increased slightly (in the moderate shock groups) until the animals died (or until the end of the experiment).

In the sham group, the blood glucose concentration was approximately 170 mg/dl (Figure 4B). In the shock groups, the blood glucose levels increased to 240 to 270 mg/dl (in moderate shock) and 300 mg/dl (in severe shock) after shock induction. However, the glucose levels decreased to approximately 200 mg/dl during the shock phase. Decreases in blood glucose continued upon resuscitation, resulting in levels of approximately 100 mg/dl. While the blood glucose concentration remained constant at this level in the moderate shock/RL group, it further decreased, to approximately 50 mg/dl, just prior to death in the moderate shock/RS group; a similar but more rapid and more pronounced decline was observed in both severe shock groups.

### Parameters of organ injury

**Figure 5:**
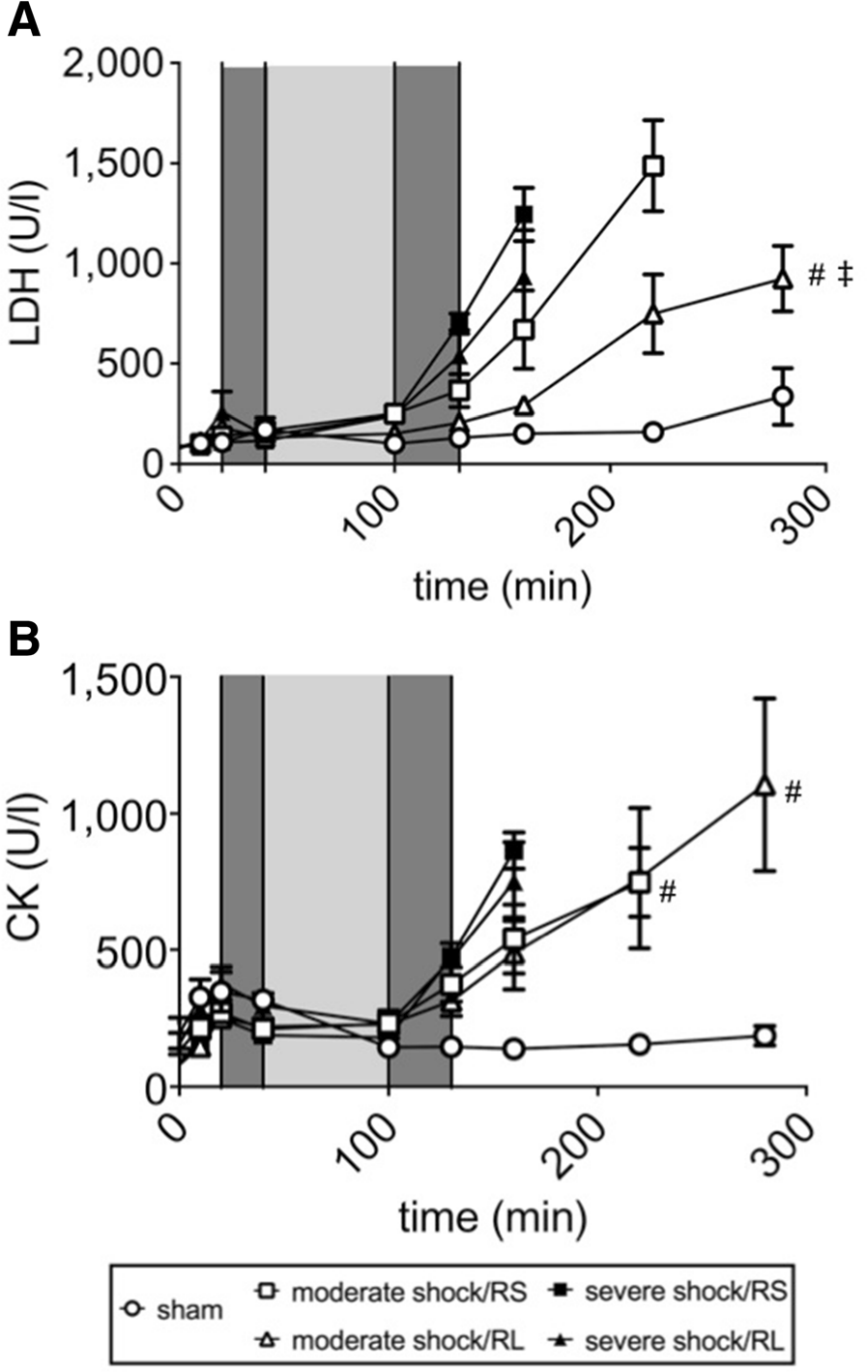
**Effects of Ringer-saline (RS) and Ringer-lactate (RL) on lactate dehydrogenase and creatine kinase activity after shock.** Rats were subjected to moderate or severe hemorrhage (shock induction: dark gray; shock phase: light gray), resuscitated (dark gray) with either Ringer-saline (RS) or Ringer-lactate (RL), and observed until the end of the experiment or until the first of the animals of the comparison groups died. Blood gas analysis was performed at distinct time points, with T = 280 minutes representing the last possible point of comparison. Lactate dehydrogenase (LDH) activity **(A)** and creatine kinase (CK) activity **(B)** were determined at the indicated time points. The values are presented as the means ± SEM (n = 8 animals per shock group, n = 6 sham group animals). SEM values that are not visible are located behind the symbols. One-way repeated-measures ANOVA and Fisher’s Least Significant Difference (LSD) *post-hoc* test were performed. # *P* <0.05 (vs. severe shock/RS group), ‡ *P* <0.05 (vs. moderate shock/RS group).

**Figure 6:**
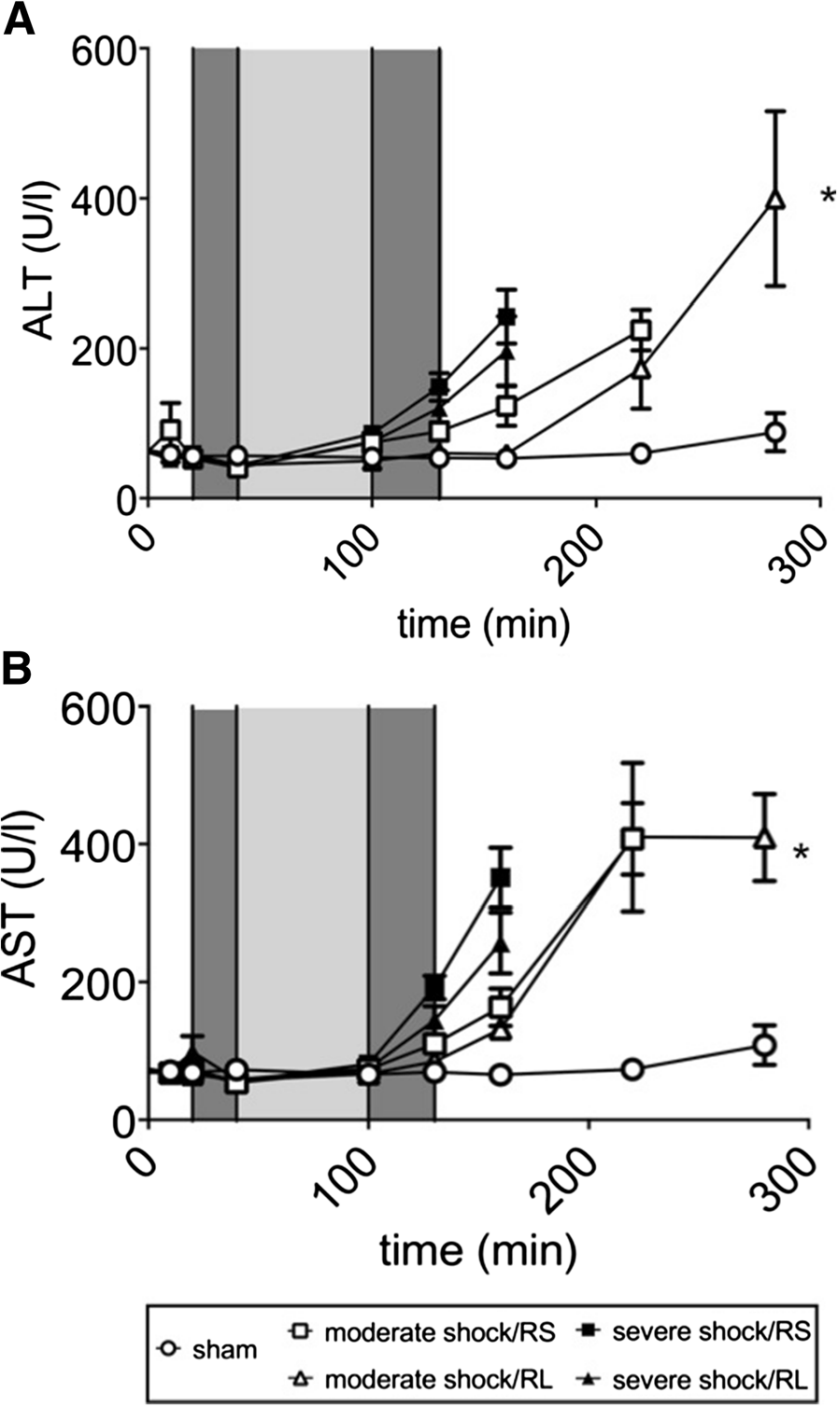
**Effects of Ringer-saline (RS) and Ringer-lactate (RL) on liver damage parameters after shock.** Rats were subjected to moderate or severe hemorrhage (shock induction: dark gray; shock phase: light gray) and then resuscitated (dark gray) with either Ringer-saline (RS) or Ringer-lactate (RL). Blood AST and ALT concentrations **(A, B)** were measured continuously throughout the experiment and recorded at the indicated time points until either the end of the experiment or the first of the animals of the comparison groups died. Blood gas analysis was performed at specific time points, with T = 280 minutes representing the last possible point of comparison. Values are presented as the means ± SEM (n = 8 animals per shock group, n = 6 sham group animals). SEM values that are not visible are located behind the symbols. One-way repeated-measures ANOVA and Fisher’s Least Significant Difference (LSD) *post-hoc* test were performed. * *P* <0.05 (vs. severe shock/RS group).

The blood plasma LDH and CK activity in the sham group animals remained below 350 U/l and 200 U/l, respectively (Figure 5A,B), and the plasma AST and ALT activities remained constant at 70 U/l and 60 U/l, respectively (Figure 6A,B). In the shock groups, all plasma enzyme activity remained at similar values during shock induction and increased only slightly, if at all, during the shock phase. Upon resuscitation and in the post-resuscitation phase, however, the plasma enzyme activities of LDH, CK, AST and ALT increased several fold. These increases were more pronounced in the severe shock animals than in the moderate shock animals. In the moderate shock/RL group; however, the increases in enzyme activities were significantly less than in the other shock groups, for example, the increase in LDH activity in the moderate shock/RL group was significantly less than the increase in the moderate shock/RS group (800 vs. 1,500 U/l at T = 240 minutes; Figure 5A).

**Figure 7:**
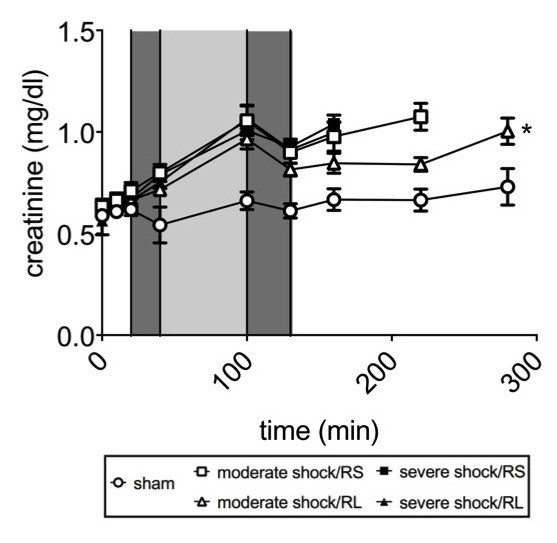
**Effects of Ringer-saline (RS) and Ringer-lactate (RL) on kidney injury parameters after shock.** Rats were subjected to moderate or severe hemorrhage (shock induction: dark gray; shock phase: light gray) and then resuscitated (dark gray) with Ringer-saline (RS) or Ringer-lactate (RL). Blood creatinine concentration was measured continuously throughout the experiment and recorded at the time points indicated until the end of the experiment or until the first of the animals of the comparison groups died. Blood gas analysis was performed at distinct time points, with T = 280 minutes representing the last possible point of comparison. Values are presented as the means ± SEM (n = 8 animals per shock group, n = 6 sham group animals). SEM values that are not visible are located behind the symbols. One-way repeated-measures ANOVA and Fisher’s Least Significant Difference (LSD) *post-hoc* test were performed. * *P* <0.05 (vs. all other shock groups).

Plasma creatinine concentrations remained stable at approximately 0.7 mg/dl in the sham group (Figure 7). During shock induction and the shock phase, plasma creatinine rose to 1 mg/dl. Upon resuscitation, plasma creatinine decreased slightly to 0.8 mg/dl and rose again after resuscitation in both severe shock groups and the moderate shock/RS group. In the moderate shock/RL group, however, plasma creatinine remained constant at 0.8 mg/l during the entire post-resuscitation phase and was thus significantly lower than the creatinine levels observed in the moderate shock/RS group.

## Discussion

Under physiological conditions, lactate is mainly produced in the skin, muscles, erythrocytes, brain and intestinal mucosa [20]. These tissues produce approximately 1,300 mmol of lactate per day. It has been reported that plasma lactate concentration may vary depending on the physiological status of the body, but to a similar extent in different species [21-23]. Up to 60% of intrinsic lactate is metabolized by the liver [20], where it is either converted back to glucose (gluconeogenesis) or degraded to CO_2_ and H_2_O. Both processes require sufficient amounts of O_2_ to be supplied to the liver; one H^+^ ion is consumed (alternative view: one HCO_3_^–^ is generated) per lactate molecule that is metabolized. Therefore, lactate is involved in maintaining the acid-base equilibrium under aerobic conditions. Hartmann first added lactate to RS to compensate for metabolic acidosis [24]. The alkalization that accompanies lactate metabolism was later confirmed by several other research groups [9,10,25,26]. In direct contrast, a recently published paper [16] and the present experiments report that neither an alkalizing effect of lactate infusion, that is, an improvement of metabolic acidosis, nor an improvement of organ injury or survival was observed in resuscitation with RL compared to resuscitation with RS in severe hemorrhagic shock. Actually, survival decreased when RL was used as a resuscitation fluid in severe shock. The lack of an effect of lactate on acid-base status most likely resulted from impaired lactate metabolism, even in the resuscitation and post-resuscitation phases. It is likely that following severe shock, distinct areas of the reperfused organs, particularly of the liver, are injured or remain anoxic despite resuscitation and are thus incapable of metabolizing lactate to glucose and/or CO_2_ and H_2_O [27]. Impaired liver function was also suggested by the dramatic drop in blood glucose concentrations that was observed in all of the shock groups. The deleterious effects of lactate infusion on survival, however, cannot solely be explained by impaired lactate metabolism in severe hemorrhagic shock. The inhibition of glycolysis by the high concentrations of lactate that accumulate under these conditions [28-30] is likely also required for these adverse effects. In line with this assumption, the lactate concentration upon resuscitation and in the post-resuscitation phase was clearly elevated in the RL group animals; although not significantly as compared with the severe shock/RS group animals.

However, in moderate hemorrhagic shock, resuscitation with RL improved metabolic acidosis and enhanced survival. Improved acid-base status is expected given the considerations given above; that is, following moderate hemorrhagic shock, lactate appears to be metabolized at a rate that is sufficiently high to consume significant amounts of H^+^ and improve the acid-base balance. Moreover, in the moderate shock, lactate appears to ameliorate gluconeogenesis, as indicated by the significantly higher blood glucose concentrations in the post-resuscitation phase, whereas in the animals resuscitated with RS blood glucose concentration dropped to a minimum. Both effects are likely to contribute decisively to the prolonged initial survival observed in the moderate shock/RL animals. Lactate infusion-related protection following moderate hemorrhagic shock is congruent with the results that have been reported by other groups in dog or swine models of moderate hemorrhagic shock [6,11].

Numerous publications and the current S3 guideline of the German Society for Trauma Surgery (Deutsche Gesellschaft für Unfallchirurgie) indicate lactated RL over normal saline (NS) for preclinical therapy in polytrauma patients with hemorrhagic shock thereby neglecting the potential adverse effects of RL [10,31,32]. However, the current guidelines do not differentiate between moderate and severe forms of hemorrhagic shock; the vast majority of trauma patients with hemorrhagic shock experience mainly moderate shock. Considering the present results, which suggest that RL is more beneficial than NS in moderate hemorrhagic shock, the general recommendation to prefer RL to NS as a resuscitation fluid appears to be justified. On the other hand, a more sophisticated recommendation to treat moderate hemorrhagic shock with RL but treat severe hemorrhagic shock with pure crystalloids, that is, non-lactated crystalloid solutions, may further improve therapeutic success. Such an approach, however, is challenging to study due to the relatively small number of patients who experience severe hemorrhagic shock and the high risk of death at the scene.

Finally, some limitations to the present study exist. The isoflurane used in the experimental model mediates vasodilation, which may maintain blood flow to vital organs but may also decrease MAP and heart rate due to vasodilatory effects [33]. However, this applies to all of the groups studied in the present experiment, and analgesia must be used for ethical reasons. More importantly, the present study uses an animal model, which cannot reflect the full scope of preclinical emergency situations. For example, it was not possible to establish preclinical permissive hypotension, which is commonly desirable, because this study focused on comparing the effects of the substances administered on different types of shock.

## Conclusions

The present study clearly demonstrates that RL is toxic in the resuscitation of severe hemorrhagic shock but protective in the treatment of moderate hemorrhagic shock with respect to initial survival, acid-base parameters and organ damage. However, further experimental studies are necessary to determine whether other metabolizable anions, such as acetate or malate, behave similarly to lactate and to elucidate the effects of lactate in permissive hypotension observed in real-life emergency situations.

## Key messages

•Ringer-lactate may mediate toxic effects in rats with severe hemorrhagic shock (mean arterial blood pressure 25 to 30 mmHg).

•Ringer-lactate is protective in the treatment of moderate hemorrhagic shock (mean arterial blood pressure 40 to 45 mmHg) with respect to initial survival.

•In moderate hemorrhagic shock, resuscitation with Ringer-lactate improved metabolic acidosis and decreased organ injury.

## Abbreviations

ACD-A: Acid citrate dextrose-A; ALT: Alanine aminotransferase; ANOVA: Analysis of variance; AST: Aspartate aminotransferase; ATLS®: Advanced Trauma LifeSupport; BE: Base excess; bpm: beats per min; CK: Creatine kinase; DGU: German Association for Trauma Surgery; HR: heart rate; LDH: Lactate dehydrogenase; LSD: Least Significant Difference; MAP: Mean arterial blood pressure; NS: Normal saline; pCO2: Carbon dioxide partial pressure; pO2: Oxygen partial pressure; RL: Ringer-lactate; RS: Ringer-saline.

## Competing interests

The authors declare that there are no competing interests.

## Authors’ contributions

BH, RR and SL conceived the study, designed the trial, and obtained research funding. SL and HdG supervised the conduct of the trial and data collection. BH and RR provided statistical advice on study design and analyzed the data. BH drafted the manuscript, and all authors contributed substantially to its revision. BH takes responsibility for the paper as a whole. All authors have read and approved the final manuscript for publication.
